# Identification of proteins differentially expressed by glutamate treatment in cerebral cortex of neonatal rats

**DOI:** 10.1186/s42826-019-0026-9

**Published:** 2019-11-26

**Authors:** Ju-Bin Kang, Dong-Ju Park, Phil-Ok Koh

**Affiliations:** Department of Anatomy, College of Veterinary Medicine, Research Institute of Life Science, Gyeongsang National University, 501 Jinju-daero, Jinju, 52828 South Korea

**Keywords:** Cerebral cortex, Glutamate, Neonate, Proteomics

## Abstract

Glutamate leads to neuronal cell damage by generating neurotoxicity during brain development. The objective of this study is to identify proteins that differently expressed by glutamate treatment in neonatal cerebral cortex. Sprague-Dawley rat pups (post-natal day 7) were intraperitoneally injected with vehicle or glutamate (10 mg/kg). Brain tissues were isolated 4 h after drug treatment and fixed for morphological study. Moreover, cerebral cortices were collected for protein study. Two-dimensional gel electrophoresis and mass spectrometry were carried out to identify specific proteins. We observed severe histopathological changes in glutamate-exposed cerebral cortex. We identified various proteins that differentially expressed by glutamate exposure. Identified proteins were thioredoxin, peroxiredoxin 5, ubiquitin carboxy-terminal hydrolase L1, proteasome subunit alpha proteins, isocitrate dehydrogenase, and heat shock protein 60. Heat shock protein 60 was increased in glutamate exposed condition. However, other proteins were decreased in glutamate-treated animals. These proteins are related to anti-oxidant, protein degradation, metabolism, signal transduction, and anti-apoptotic function. Thus, our findings can suggest that glutamate leads to neonatal cerebral cortex damage by regulation of specific proteins that mediated with various functions.

## Introduction

Glutamate acts as a critical excitatory neurotransmitter in central nervous system [1]. It plays an important role in synaptic maintenance and plasticity, learning and memory, and cytoskeleton formation [2]. However, excessive glutamate exposure induces excitotoxicity and apoptosis, causes neuronal dysfunction [3]. Glutamate excitotoxicity leads to neurodegenerative disorders, such as epilepsy and Alzheimer’s disease [4]. Moreover, excessive glutamate release accelerates calcium influx into intracellular matrix through glutamate receptor. Increased calcium concentration induces severe mitochondrial damage and consequently leads to cell death [5, 6]. Glutamate excitotoxicity in neonate produces a pathophysiological impact on adulthood. It changes blood-brain barrier permeability, increases neurovascular permeability, and results in neuronal cell death [7]. Glutamate treatment induces an excitotoxic neurodegenerative process that associated with pathological conditions during postnatal development. Moreover, glutamate also modifies vascular endothelial growth factor (VEGF) and its receptor expression in neonatal cerebral cortex, consequently leads to various neuropathological conditions [8]. It is well accepted that VEGF exerts neuroprotective and pro-inflammatory effects during brain development. Glutamate induces oxidative stress in neonatal brain regions and increases nociceptive behavior by thermal stimuli and mechanical allodynia [9]. We propose that glutamate treatment in neonate induces neuropathological changes by modulating various proteins. Thus, we investigated specifically regulated proteins by glutamate treatment in neonatal cerebral cortex using a proteomics technique.

## Materials and methods

### Experimental animals and drug administration

Pregnant female Sprague-Dawley rats were purchased from Samtako Co. (Animal Breeding Centre, Osan, Korea) to obtain pups. Rats were kept in controlled temperature (25 °C) and lighting (12 h:12 h light/dark cycle) conditions with free access to feed and water. All experimental procedures were performed in accordance with the provided guidelines by the Institutional Animal Care and Use Committee of Gyeongsang National University. Pups at post-natal 7 day were randomly divided into two groups, vehicle- and glutamate-treated groups (*n* = 8 per group). Glutamate (10 mg/kg, Sigma, St. Louis, MO, USA) was dissolved in normal saline and intraperitoneally injected. Vehicle-treated animals were administrated with only normal saline. Pups were sacrificed 4 h after treatment and separated brain from skull were fixed in 4% paraformaldehyde in 0.1% phosphate-buffered saline (pH 7.4) for morphological study. Cerebral cortices were isolated from brain and immediately frozen in liquid nitrogen for proteomics study.

### Hematoxylin and eosin staining

Fixed tissues were washed with tap water, dehydrated with gradient ethanol series from 70 to 100%, cleaned with xylene, and embedded in paraffin using embedding center (Leica, Westlar, Germany). Paraffin blocks were sliced into 4 μm thickness using a rotary microtome (Leica) and paraffin ribbons were placed on slide glass. Tissues were deparaffinized with xylene, rehydrated with gradient ethanol series from 100 to 70%, and immersed in water. They were stained with Harris’ hematoxylin solution (Sigma) for 3 min, washed with tap water for 10 min, stained with eosin Y solution for 1 min, and dipped with water. They were dehydrated with gradient ethanol series from 70 to 100%, cleaned with xylene, and mounted with permount solution (Thermo Fisher Scientific, Waltham, MA, USA). Stained tissues were observed under Olympus microscope (Olympus, Tokyo, Japan) and microscopic images were taken. Total neurons and damaged neurons were counted in five random areas (500 × 500 μm) and percentage of damaged neurons was calculated by following formula: The number of damaged neurons / Total neurons × 100.

### 2-dimensional gel electrophoresis

Cerebral cortices were homogenized in lysis buffer (8 M urea, 4% CHAPS, 0.2% ampholyte, 40 mM Tris-HCl), centrifuged at 20,000 g for 20 min at 4 °C, and supernatant was collected. Proteins were precipitated with 10% trichloroacetic acid for 30 min and centrifuged at 20,000 g for 20 min at 4 °C again to condense the proteins. Proteins pellets were washed with 1 M Tris-HCl (pH 7.6) and dried at room temperature. Dried protein pellets were dissolved in sample buffer [8 M urea, 4% CHAPS, 0.2% ampholyte, 40 mM Tris-HCl, 2 μg/ml dithiothreitol (DTT)], sonicated for 3 min, incubated for 1 h at room temperature, and centrifuged at 15,000 g for 30 min at 4 °C. Supernatants were collected and protein concentrations were determined using Bradford protein assay kit (Bio-Rad, Hercules, CA, USA) according to the manufacturer’s protocol. The immobilized pH gradient (IPG) gel strips (17 cm, pH 4–7 and pH 6–9; Bio-Rad) were used for first dimensional isoelectric focusing. Strips were incubated with rehydration solution (8 M urea, 2% CHAPS, 20 mM DTT, 0.5% IPG buffer, bromophenol blue) for overnight at room temperature. Strips were placed in sample cup containing 50 μg protein sample and isoelectric focusing was performed using the Ettan IPGphor 3 system (GE Healthcare, Uppsala, Sweden) by following conditions: 250 V for 15 min, 10,000 V for 3 h, and 10,000 to 50,000 V. They were incubated with equilibration buffer (6 M urea, 30% glycerol, 2% sodium dodecyl sulfate, 50 mM Tris-HCl, bromophenol blue) containing 1% DTT for 10 min and reacted with equilibration buffer containing 2.5% iodoacetamide for 10 min. IPG gel strips were loaded into 7.5–17.5% gradient gel and second dimensional separation was carried out in 10 mA at 10 °C using a Protein-II XI electrophoresis equipment (Bio-Rad). Electrophoresis was continued until the bromophenol blue dye reached to the bottom. Gels were fixed in fixing solution (12% acetic acid in 50% methanol) for 2 h, washed with 50% ethanol for 20 min, kept in sodium thiosulfate for 1 min, and washed with distilled water for 1 min. They were stained with silver stain solution (0.2% silver nitrate, 0.03% formaldehyde) for 20 min, washed with distilled water for 1 min, and reacted with developing solution (2% sodium carbonate, 0.02% formaldehyde) until protein spots were clearly stained. Gels were incubated with stop solution (1% acetic acid) and scanned using Agfar ARCUS 1200™ (Agfar-Gevaert, Mortsel, Belgium). Stained protein spots were analyzed with PDQuest 2-DE analysis software (Bio-Rad). Changed proteins between vehicle- and glutamate-treated groups were investigated. Identified specific protein spots were cut from gels, reacted with destained solution (30 mM potassium hexacyanoferrate, 100 mM sodium thiosulfate), and washed with washing solution (10% acetic acid in 50% methanol) for destaining silver stain. They were dehydrated with 50 mM ammonium bicarbonate and acetonitrile, and dried in vacuum centrifuge for 20 min. They were incubated with reduction solution (10 mM DTT in 0.1 M ammonium bicarbonate) at 56 °C for 45 min, dehydrated with 0.1 M ammonium bicarbonate and acetonitrile, and dried in vacuum centrifuge for 20 min. Dried spots were reacted with a digestion solution (12.5 ng/ml trypsin, 0.1% octyl beta-D glycopyranside in 50 mM ammonium bicarbonate) overnight at 37 °C and incubated with extraction buffer (1% trifluoroacetic acid in 66% acetonitrile) to collect digested proteins. Extracted proteins were finally dehydrated in a vacuum centrifuge for 2 h. Matrix solution was prepared by dissolving alpha-cyano-4-hyroxycinnamic acid and nitrocellulose in acetone. Dried proteins were dissolved in extraction buffer and matrix solution, and loaded on a matrix-assisted laser desorption ionization-time (MALDI-TOF) plate. MALDI-TOF was performed with Voyager-DE STR (Applied Biosystem, Foster City, CA, USA) and the peak of obtained results was analyzed through NCBI and MS-FIT protein sequence database.

### Statistical analysis

Results were presented as the mean ± standard error of means (S.E.M.). The results of each group were compared by one-way analysis of variance (ANOVA) followed by *post-hoc* Scheffe’s test. *P* < 0.05 was regarded as statistically significant.

## Results

**Fig. 1:**
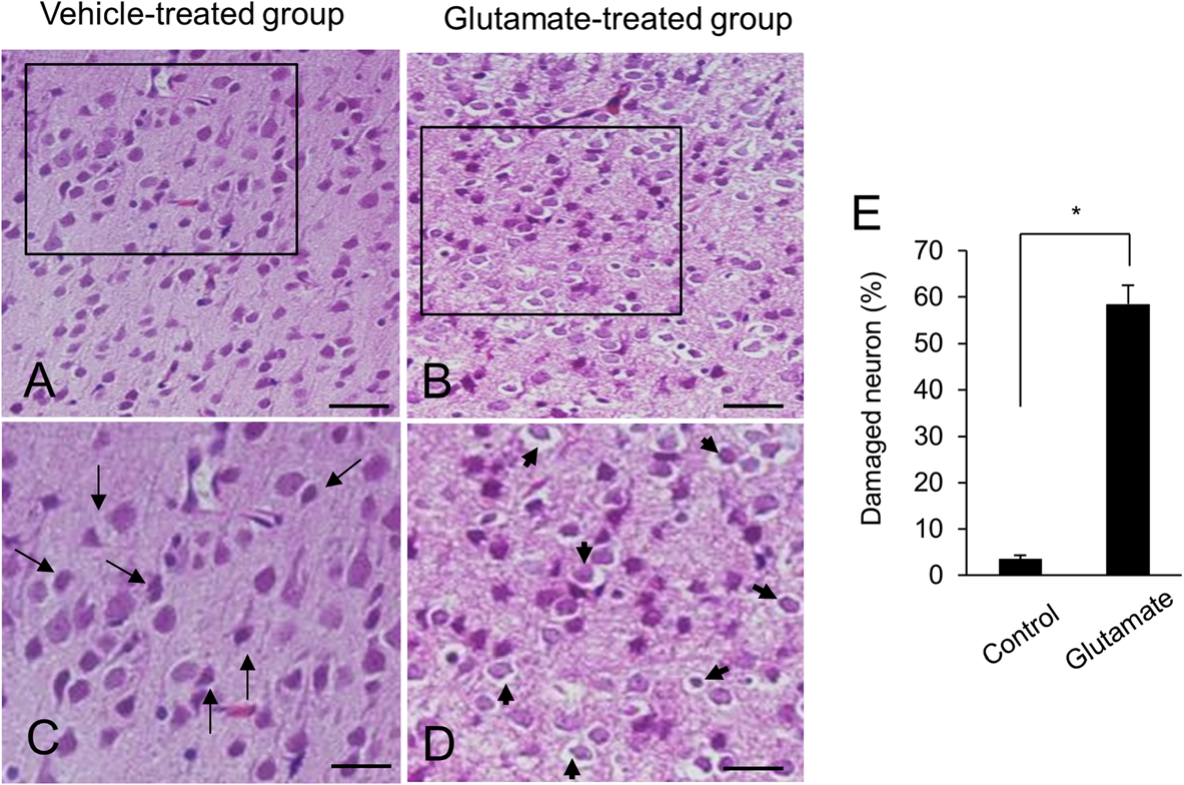
Representative photomicrographs of hematoxylin and eosin staining in neonatal cerebral cortices of vehicle- (**a** and **c**) and glutamate-treated (**b** and **d**). **c** and **d** photos are the magnification of **a** and **b** squares. Arrows indicate normal neurons with well-developed dendrites and typical pyramidal shape. Arrowheads indicate damaged neurons with shrunken dendrites and untypical round shape. Glutamate treatment significantly increased the percentage of damaged neuron in cerebral cortex (**e**). Scale bar: 100 μm (**a** and **b**), 50 μm (**c** and **d**). Data (*n* = 4) are shown as mean ± S.E.M. * *P* < 0.05

**Fig. 2:**
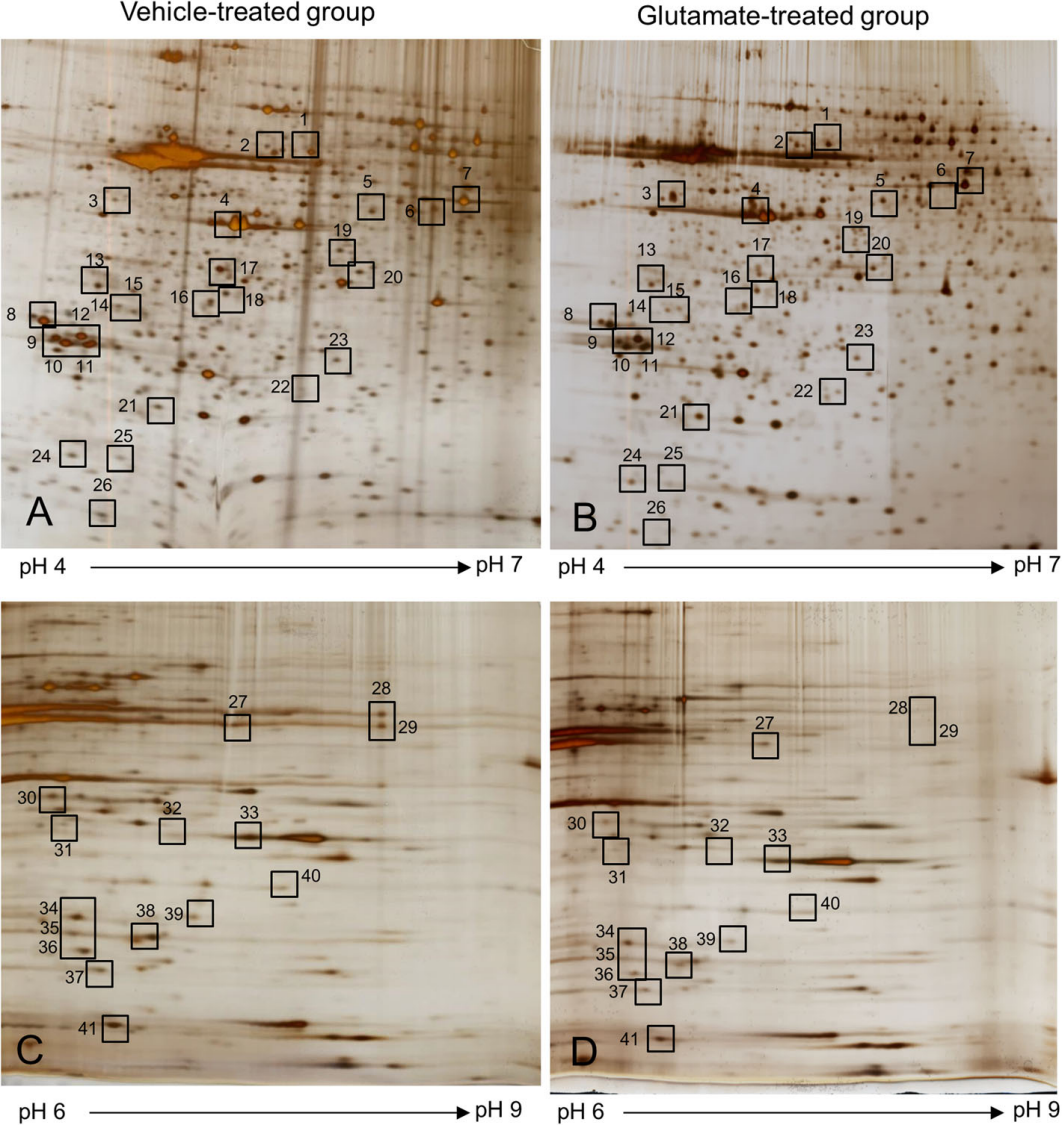
Two-dimensional electrophoresis analysis of proteins in neonatal cerebral cortices in vehicle- (**a** and **c**) and glutamate- (**b** and **d**) treated animals. Isoelectric focusing was performed at pH 4–7 (**a** and **b**) and pH 6–9 (**c** and **d**) using IPG strips, followed by second dimensional separation on 7.5–17.5% gradient SDS gels stained with silver nitrate. Squares indicate the protein spots with more than two folds intensity change between vehicle- and glutamate-treated animals

**Fig. 3:**
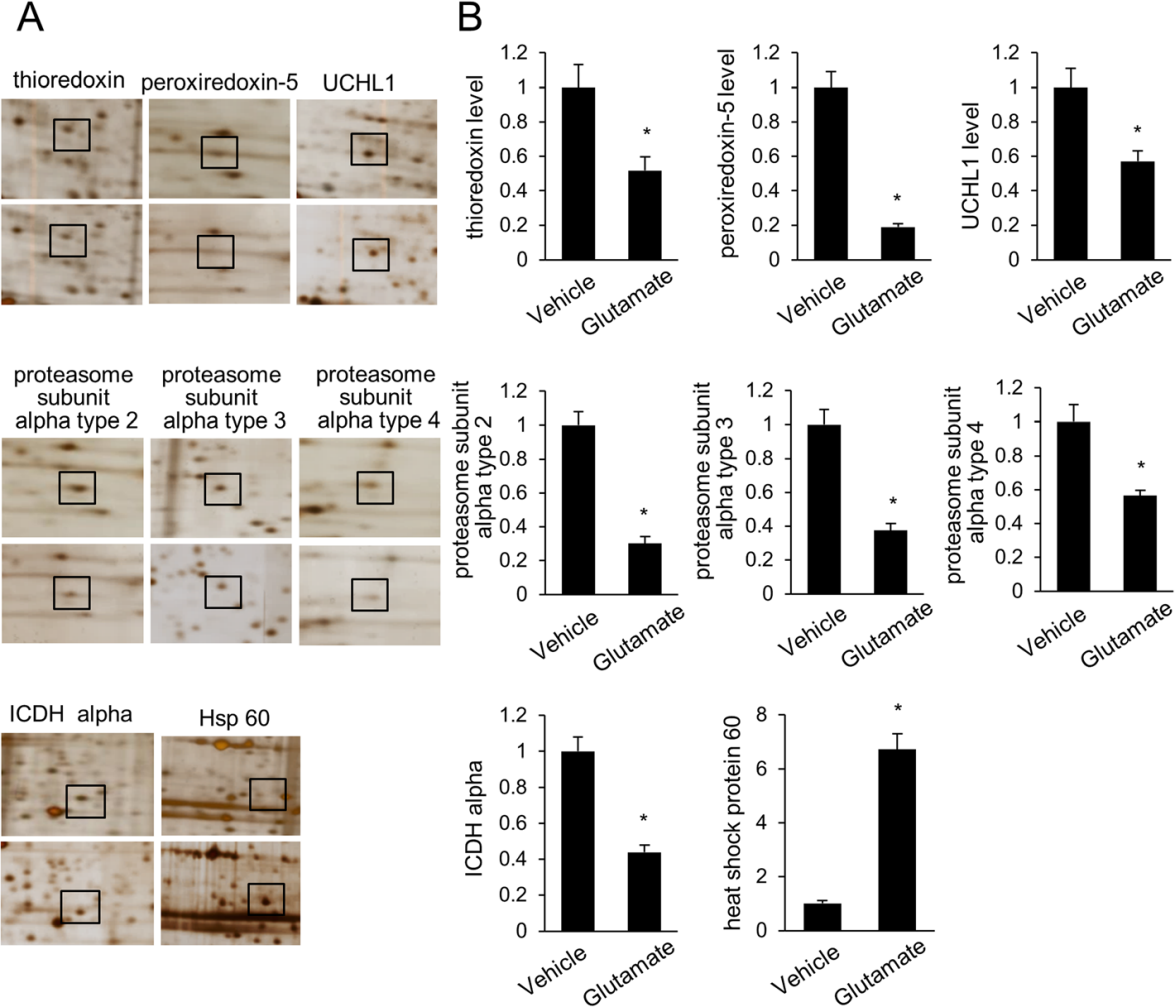
Magnified protein spots of thioredoxin, peroxiredoxin 5, ubiquitin carboxy-terminal hydrolase L1 (UCHL1), proteasome subunit alpha type 2, 3, 4, isocitrate dehydrogenase (ICDH) alpha, and heat shock protein 60 in neonatal cerebral cortices of vehicle- and glutamate-treated animals (**a**). Squares indicate these protein spots. Spot intensities were measured by PDQuest software and are reported as a ratio relative to vehicle-treated animals (**b**). Data (*n* = 4) are shown as mean ± S.E.M. * *P* < 0.05

**Fig. 4:**
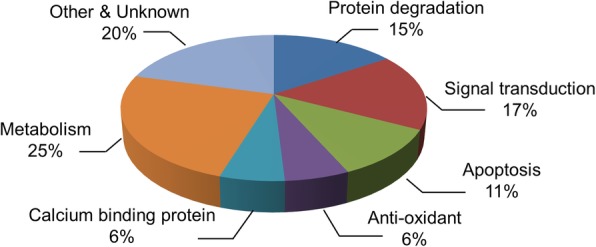
A schematized pie chart of classified proteins according to function in differentially expressed protein by glutamate exposure in neonatal cerebral cortex

Figure 1 showed the representative photos of hematoxylin and eosin staining. We observed the histopathological changes of cerebral cortex in glutamate-treated animals. Vehicle-treated animals had neurons with well-developed dendrites and typical pyramidal shape cell body with round and large nucleus (Fig. 1a and c). However, glutamate-treated animals had neurons with shrunken dendrites and untypical round shape cell body (Fig. 1b and d). Percentage of damaged neurons was significantly increased by glutamate treatment (Fig. 1e). Percentages of damaged neurons were 3.55 ± 0.77 and 58.48 ± 4.11 in control animals and glutamate-treated animals, respectively. Figure 2 showed the images of two-dimensional electrophoresis maps in the range pH 4–7 and pH 6–9 for the cerebral cortices of vehicle- and glutamate-treated animals. Approximately 850 and 390 protein spots were detected in pH 4–7 and pH 6–9 images, respectively (Fig. 2). We found forty-one protein spots with a more than two-fold intensity change between vehicle- and glutamate-treated animals. Among these proteins, thirty-six protein spots were identified by MALDI-TOF analysis (Table 1). Most of identified proteins were decreased in glutamate-treated animals compared to those of vehicle-treated animals. However, heat shock protein 60 level was significantly increased in glutamate-treated animals. Moreover, three unknown proteins were decreased in glutamate-treated animals, while two unknown proteins were increased. Figure 3a showed magnified protein spots of thioredoxin, peroxiredoxin 5, ubiquitin carboxy-terminal hydrolase L1, proteasome subunit alpha type 2, 3, 4, isocitrate dehydrogenase, and heat shock protein 60. Identified protein level was expressed as a ratio of the intensity in glutamate-treated animals to the intensity in vehicle-treated animals (Fig. 3b). Thioredoxin and peroxiredoxin 5 levels were 0.52 ± 0.08 and 0.19 ± 0.02 in glutamate-treated animals, respectively. Ubiquitin carboxy-terminal hydrolase L1 and proteasome subunit alpha type 2 levels were 0.57 ± 0.06 and 0.30 ± 0.04 in glutamate-treated animals. Proteasome subunit alpha type 3 and 4 levels were 0.38 ± 0.04 and 0.57 ± 0.03 in glutamate-treated animals. Isocitrate dehydrogenase and heat shock protein 60 levels were 0.44 ± 0.04 and 6.72 ± 0.58 in glutamate-treated animals, respectively. Identified proteins were classified according to various functions, including anti-oxidant, protein degradation, metabolism, and anti-apoptosis. Thioredoxin and peroxiredoxin 5 are anti-oxidant proteins. Protein degradation related proteins are ubiquitin carboxy-terminal hydrolase L1 and proteasome subunit alpha type 2, 3, 4. Metabolism related proteins are isocitrate dehydrogenase (NAD+) subunit alpha, succinyl-CoA ligase subunit beta, glyceraldehyde-3-phosphate dehydrogenase, and adenylate kinase isoenzyme 1. Signal transduction related proteins are adenosylhomocysteinase, alpha-synuclein, and mitogen-activated protein kinase. Apoptosis related proteins are 14–3-3 family proteins. Figure 4 represented a schematized pie chart of classified proteins according to their functions.

**Table 1:** List of proteins that were differentially expressed between vehicle- and glutamate-treated animals

Spot no	Protein name	Accession no	Mw (kDa)	pI	Mass matched	Sequence Coverage (%)
Protein degradation						
2	heat shock protein 60	P63038	60.96	5.91	19/45	41
13	ubiquitin carboxy-terminal hydrolase L1	Q7TQI3	31.27	4.85	11/66	58
14	ubiquitin thiolesterase OTUB1	B2RYG6	31.27	4.8	14/39	61
23	proteasome subunit alpha type 3	P18422	28.40	5.3	7/112	27
36	proteasome subunit alpha type 2	P17220	25.93	6.9	6/109	33
39	proteasome subunit alpha type 4	P21670	29.50	7.6	5/98	22
Signal transduction						
1	dihydropyrimidinase-related protein 2	P47942	62.27	6.0	24/103	52
5	rab GDP dissociation inhibitor beta	P50399	50.50	5.9	9/150	23
7	adenosylhomocysteinase	P10760	47.54	6.07	15/132	33
18	protein phosphatase 2A, subunit A	P63331	35.60	5.3	13/110	45
24	alpha-synuclein	P37377	14.50	4.7	6/134	43
30	mitogen-activated protein kinase 1	P63086	41.28	6.5	10/96	36
31	mitogen-activated protein kinase 1	P63086	41.28	6.5	10/96	36
Apoptosis						
8	14–3-3 Gamma	P61983	28.28	4.8	8/77	35
9	14–3-3 Zeta/delta	P63102	27.77	4.7	12/122	41
10	14–3-3 Zeta/delta	P63102	27.77	4.7	8/125	36
11	14–3-3 Beta/alpha	P35213	28.05	4.8	11/110	43
12	14–3-3 Epsilon	P62260	29.17	4.6		
Anti-oxidant						
15	thioredoxin	Q920J4	32.23	4.84	8/87	42
35	peroxiredoxin-5	Q9R063	22.17	8.9	9/114	46
Calcium binding protein						
25	hippocalcin	P62749	22.32	5.3	10/102	52
26	parvalbumin alpha	P02625	11.92	5	5/103	40
Metabolism						
6	succinyl-CoA ligase subunit beta, mitochondrial	Q9Z2I9	50.11	6.6	6/104	12
20	isocitrate dehydrogenase (NAD+) subunit alpha	Q99NA5	39.59	6.47	8/93	31
27	pyruvate kinase isoenzyme M1/M2	P11980	57.82	6.63	19/80	42
32	glyceraldehyde-3-phosphate dehydrogenase	P04797	35.83	8.14	16/106	46
33	glyceraldehyde-3-phosphate dehydrogenase	P04797	35.83	8.14	21/94	62
34	nucleoside diphosphate kinase B	P19804	17.28	6.9	8/86	49
37	adenylate kinase isoenzyme 1	P39069	21.58	7.7	6/113	34
38	triosephosphate isomerase	P48500	26.85	6.9	11/103	51
40	vacuolar proton pump subunit E1	Q6PCU2	26.13	8.4	11/101	40
41	nucleoside diphosphate kinase B	P19804	17.28	6.9	8/86	49
Other & Unknown						
4	prolactin-8A5 isoform XI	P33579	27.27	5.47	7/97	22
16	mu-crystallin	Q9QYU4	33.53	5.34	9/86	24
17	mu-crystallin	Q9QYU4	33.55	5.3	6/114	21
3	unknown					
19	unkonwn					
21	unknown					
28	unknown					
29	unknown					

Protein names and accession numbers are listed according to the SWISS-PROT database. *Mw* Molecular weight, *pI* Isoelectric point

## Discussion

Glutamate is a major excitatory neurotransmitter that released in neurons and astrocyte. Glutamate excitotoxicity leads to neonatal brain damage and neurodegenerative disorder. Moreover, overexposure of glutamate induces oxidative stress and leads to neuronal cell death [10, 11]. We confirmed histopathological changes in glutamate-treated neonatal cerebral cortex. We also identified specifically regulated proteins by glutamate exposure in neonatal cerebral cortex using a proteomics approach. We classified these proteins according to proteins function. These proteins are related with anti-oxidant, protein degradation, signal transduction, metabolism, and apoptosis. Among these proteins, we discussed on thioredoxin, peroxiredoxin 5, ubiquitin carboxy terminal hydrolase L1, proteasome subunit alpha, isocitrate dehydrogenase, and heat shock protein 60.

Redox regulation is important process for defense system from oxidative stress in neuron. Excitotoxic insults reduce thioredoxin activity, induce peroxiredoxin hyperoxidation, increase oxidative stress, and result in neuronal cell damage. Thioredoxin is a redox protein that prevents peroxiredoxin hyperoxidation and enhances resistance to oxidative stress [12, 13]. Overexpression of thioredoxin decreases neuronal cell damage and preserves neurons from oxidative stress [12, 13]. Peroxiredoxin 5 is an anti-oxidant enzyme that decreases hydrogen peroxide and alkyl hydroperoxide. It plays a critical role in cellular homeostasis by scavenging cellular ROS and ameliorates glutamate-induced apoptosis in neuronal cells culture [14, 15]. Moreover, knockdown of peroxiredoxin 5 increases apoptosis caused by glutamate exposure [16]. Peroxiredoxin 5 exerts a neuroprotective effect against excitotoxic brain injury in newborn mice [17]. We showed a decrease of peroxiredoxin 5 in glutamate-treated neonatal cerebral cortex. A decrease of peroxiredoxin 5 indicates the induction of apoptotic cell death. We also showed a decrease of thioredoxin expression caused by glutamate in neonatal cerebral cortex. Thioredoxin-peroxiredoxin system contributes to protective effect against oxidative stress. Down-regulation of thioredoxin and peroxiredoxin 5 weak resistance to oxidative stress and lead to cell death. Thus, we demonstrate that glutamate excitotoxicity leads to thioredoxin and peroxiredoxin 5 decreases, and finally induces neuronal cell damage during postnatal development.

Ubiquitination and deubiquitination of proteins are essential processes for the maintenance of cell homeostasis. Ubiquitin carboxy terminal hydrolase L1 abundantly exists in neuron, which ubiquitinates damaged proteins, and activates the ubiquitin proteasome system [18, 19]. Ubiquitin carboxy terminal hydrolase L1 decreases in neurodegenerative deficits on both adult and childhood [20, 21]. Therefore, ubiquitin carboxy terminal hydrolase L1 is considered as a biomarker for neuronal cell death [22]. Our results demonstrated that glutamate decreases ubiquitin carboxy terminal hydrolase L1 expression in neonatal cerebral cortex. A decrease of ubiquitin carboxy terminal hydrolase L1 leads to an imbalance between ubiquitination and deubiquitination, results in neuronal cell death [23]. Thus, this result can suggest that glutamate exposure regulates ubiquitin carboxy terminal hydrolase L1 expression, accumulates aggregated proteins, and induces neuronal cell death in neonatal cerebral cortex.

Proteasomal degradation pathway is very important for various cellular processes, including cell cycle, gene expression regulation, and oxidative stress response. Proteasomes are protein complexes that remove damaged or unneeded proteins by proteolysis. Misfolded proteins were degraded by tagging with a single ubiquitin molecule. Thus, ubiquitination and proteosomal degradation is an essential cellular proteolytic system to remove oxidative proteins. Oxidative stress induces proteasome inhibition and leads to cell degradation and apoptotic cell death. Moreover, ischemic injury accumulates oxidized proteins and decreases proteasome activity. We identified decreases of proteasome subunit alpha proteins in glutamate-exposed neonatal cerebral cortex. Proteasome subunit alpha proteins are involved in proteolytic degradation of most intracellular proteins [24]. Glutamate treatment induces DNA damage by increasing reactive oxygen species [25]. Moreover, damaged DNA decreases proteasome subunit alpha expression [26]. A decrease of proteasome induces cellular dysfunction and cellular alteration [27]. Thus, we demonstrate that glutamate toxicity leads to proteasome proteins decreases and finally induces cerebral cortex damage during postnatal development.

Isocitrate dehydrogenase is a metabolic enzyme that plays important roles in tricarboxylic acid cycle [28]. It reduces NAD+ to NADH by catalyzes isocitrate to α-ketoglutarate and CO_2_ [29]. This metabolic reaction is an essential event for ATP production and lack of this enzyme causes a serious metabolic dysfunction [30]. Isocitrate dehydrogenase abundantly exists in neurons, microglia, and astrocytes [31]. It is down-regulated in Alzheimer’s disease and stroke [32, 33]. We showed a decrease of isocitrate dehydrogenase in glutamate-treated neonatal cerebral cortex. A decrease of isocitrate dehydrogenase induces metabolic disorders. Thus, we can suggest that glutamate excitotoxicity decreases isocitrate dehydrogenase expression, causes the metabolic dysfunctions, and finally induces neuronal cell damage.

Heat shock protein 60 is a mitochondrial protein that involved in protein folding, transport, and protection [34]. It inhibits protein misfolding, maintains mitochondrial proteostasis, and prevents protein damage [34]. Heat shock protein 60 expression increases under oxidative stress condition [35]. Increased heat shock protein 60 leads to neurodegeneration in neonatal brain by inducing immune response [36]. Increase of heat shock protein 60 indicates damaged condition. We detected an increase of heat shock protein 60 in glutamate-exposed neonatal cerebral cortex. Thus, we confirmed that glutamate excitotoxicity induces an increase of heat shock protein 60 in neonatal cell damage caused by glutamate. In summary, glutamate induces neuronal cell damage by modulating several proteins in neonatal cerebral cortex. This study demonstrates that glutamate exposure decreases thioredoxin, peroxiredoxin 5, ubiquitin carboxy terminal hydrolase L1, proteasome subunit alpha proteins, and isocitrate dehydrogenase levels. In contrast, heat shock protein 60 level is increased by glutamate treatment. Our results provide evidence that glutamate exposure induces damages by modulating specific proteins in neonatal cerebral cortex neuronal cell.

## Conclusion

Results of this study showed that glutamate exposure induces neuronal damage in neonatal cerebral cortex by the regulation of specific proteins that related to various functions. Therefore, our findings may suggest a valuable evidence for understanding the mechanism of neuronal cell damage caused by glutamate treatment during postnatal development.

## Data Availability

The data that support the findings of this study are available on request from the corresponding author on reasonable request.

## Abbreviations

DTT: Dithiothreitol
IPG: Immobilized pH gradient
MALDI-TOF: Matrix-assisted laser desorption ionization-time
NRF: National research foundation of Korea
VEGF: Vascular endothelial growth factor
